# Ataxia with oculomotor apraxia type 2 caused by a novel homozygous mutation in SETX gene, and literature review

**DOI:** 10.3389/fnmol.2022.1019974

**Published:** 2022-11-10

**Authors:** Shuaishuai Chen, Juping Du, Huihua Jiang, Weibo Zhao, Na Wang, Anna Ying, Jun Li, Shiyong Chen, Bo Shen, Yuanlin Zhou

**Affiliations:** ^1^Department of Clinical Laboratory, Taizhou Hospital of Zhejiang Province Affiliated to Wenzhou Medical University, Linhai, China; ^2^Department of Neurology, Taizhou Hospital of Zhejiang Province Affiliated to Wenzhou Medical University, Linhai, China; ^3^Department of Orthopedics, Taizhou Hospital of Zhejiang Province Affiliated to Wenzhou Medical University, Linhai, China

**Keywords:** ataxia with oculomotor apraxia type 2, *SETX* gene, early-onset menopause, Whole-exome sequencing, clinical and genetic spectrum

## Abstract

**Objectives:**

Autosomal recessive inherited ataxia with oculomotor apraxia type 2 (AOA2), caused by *SETX* gene mutations, is characterized by early-onset, progressive cerebellar ataxia, peripheral neuropathy, oculomotor apraxia and elevated serum α-fetoprotein (AFP). This study aimed to expand and summarize the clinical and genetic characteristics of *SETX* variants related to AOA2.

**Methods:**

The biochemical parameters, electromyogram and radiological findings of the patient were evaluated. Whole-exome sequencing (WES) was performed on the patient using next-generation sequencing (NGS), the variants were confirmed by Sanger sequencing and the pathogenicity of the variants was classified according to the American College of Medical Genetics and Genomics/Association for Molecular Pathology (ACMG/AMP) guidelines. We reviewed 57 studies of AOA2 patients with *SETX* mutations and collected clinical and genetic information.

**Results:**

The patient was a 40-year-old Chinese woman who primarily presented with numbness and weakness of the lower limbs in her teenage years. She had elevated AFP, increased serum follicle-stimulating hormone (FSH) and luteinizing hormone (LH) and decreased anti-Müllerian hormone (AMH) levels. We identified a novel homozygous missense mutation of the *SETX* gene, c.7118 C>T (p. Thr2373Ile), in the patient via Whole-exome and Sanger sequencing. The variant was located in the DNA/RNA helicase domain and is highly conserved. The protein prediction analysis verified the *SETX* variant as a damaging alteration and ACMG/AMP guidelines classified it as likely pathogenic. Through a literature review, we identified 229 AOA2 cases with *SETX* variants, and among the variants, 156 *SETX* variants were exonic. We found that 107 (46.7%) patients were European, 50 (21.8%) were African and 48 (21.0%) were Asian. Among the Asian patients, five from two families were Mainland Chinese. The main clinical features were cerebellar ataxia (100%), peripheral neuropathy (94.6%), cerebellar atrophy (95.3%) and elevated AFP concentration (92.0%). Most reported *SETX* mutations in AOA2 patients were missense, frameshift and nonsense mutations.

**Conclusion:**

We discovered a novel homozygous variant of the *SETX* gene as a cause of AOA2 in the current patient and expanded the genotypic spectrum of AOA2. Moreover, the clinical features of AOA2 and genetic findings in *SETX* were assessed in reported cohorts and are summarized in the present study.

## Introduction

Hereditary autosomal recessive cerebellar ataxias (ARCAs) are neurodegenerative diseases with many different phenotypes (Le Ber et al., 2005; Chiang et al., 2022). Ataxia with oculomotor apraxia type 2 (AOA2) (also known as spinocerebellar ataxia with axonal neuropathy-2 (SCAN2), Online Mendelian Inheritance of Man (OMIM) #606002) is one type of ARCA with a worldwide distribution. The main clinical manifestations of AOA2 include early-onset, progressive cerebellar ataxia, peripheral axonal sensorimotor neuropathy, oculomotor apraxia (OMA) and cerebellar atrophy. Laboratory assays of AOA2 patients frequently reveal elevated serum α-fetoprotein (AFP) concentrations (Anheim et al., 2009; Nanetti et al., 2013).

AOA2 is caused by mutations in the *SETX* gene (OMIM #608465) located on chromosome 9q34 (Nanetti et al., 2013). Human *SETX* encodes a 2677-amino acid protein named senataxin, which was mainly located in the nucleoplasm, and contains an N-terminal protein interaction domain and a DNA/RNA helicase in the C-terminal (Moreira et al., 2004; Suraweera et al., 2007; Yuce and West, 2013). As a large DNA/RNA putative helicase protein, senataxin shows homology to the yeast splicing endonuclease 1 protein (Sen1p) (Moreira et al., 2004). Senataxin is involved in the regulation of transcription, protecting genome integrity against the DNA damage response and oxidative stress and autophagy (Tariq et al., 2018; Richard et al., 2021). Moreover, mutations in the *SETX* gene are also associated with an autosomal dominant inheritance form of the juvenile amyotrophic lateral sclerosis 4 (ALS4) (OMIM 602433).

In this study, we report the clinical and genetic characterizations of an AOA2 patient with early menopause. A new homozygous mutation in *SETX* was identified in the patient. To the best of our knowledge, there were few reports about AOA2 in Mainland China. Additionally, we reviewed 57 studies that reported AOA2 patients with *SETX* mutations from 2004 to 2022 to identify the clinical and genetic spectrum of AOA2 caused by *SETX* mutations and summarize the relevant clinical symptoms of AOA2 patients.

## Materials and methods

### Ethics statement

The study was approved by the Ethics Committee of Taizhou Hospital of Zhejiang Province Affiliated to Wenzhou Medical University. The family members signed informed consent prior to sample collection and gene testing.

### Study subjects and clinical examinations

Clinical assessment of the proband and her parents was performed by neurologists, including physical and neurological examinations. The proband was considered to have hereditary peripheral neuropathy, as she had progressive gait disturbance and muscle atrophy. The blood samples and pedigree were collected from the family after obtaining informed consent. The levels of laboratory indicators, such as serum α-fetoprotein (AFP), creatine kinase (CK), albumin, immunoglobulin, follicle-stimulating hormone (FSH), luteinizing hormone (LH) and anti-Müllerian hormone (AMH), were measured. Electromyography (EMG) and nerve conduction studies (NCSs) were evaluated. Brain magnetic resonance imaging (MRI) of the proband was performed on a GE Signa TwinSpeed 1.5T Medical System. Cognitive function was assessed using the Montreal Cognitive Assessment (MoCA) and Mini-Mental State Examination (MMSE) by neuro physicians.

### Genetic testing and data analysis

Genomic DNA was isolated from the peripheral blood of the proband and her parents and used for DNA library preparation. Sequencing library construction was performed using Agilent SureSelect Human All ExonV6 kits (60 M capture, Agilent Technologies, Santa Clara, CA, USA) according to the manufacturer's instructions. Products were purified by an AMPure XP system (Beckman Coulter, Beverly, MA, USA) and quantified on an Agilent 2100 system. Whole-exome sequencing (WES) was performed by using next-generation sequencing (NGS) techniques on an Illumina NovaSeq 6000 Sequencing System with 150 bp paired-end sequences. The DNA sequence was referred to the UCSC hg19/GRCh37 (University of California Santa Cruz version hg19/Genome Reference Consortium Human Build 37) human reference sequence, and alignment analysis was performed by Burrows–Wheeler Aligner (BWA) v0.7.1. The variation annotation database includes RfeSeq (NCBI Reference Sequence Database) (https://www.ncbi.nlm.nih.gov/refseq/rsg/), Single Nucleotide Polymorphism Database (dbSNP, https://www.ncbi.nlm.nih.gov/snp/), 1000 Genomes Project (https://www.internationalgenome.org/) and the Genome Aggregation Database (gnomAD, http://gnomad-sg.org/). The pathogenicity and clinical significance of the variants were analyzed using databases such as ClinVar (https://www.ncbi.nlm.nih.gov/clinvar/), the Human Gene Mutation Database (HGMD, https://www.hgmd.cf.ac.uk/ac/index.php) and OMIM (Online Mendelian Inheritance in Man, https://www.omim.org/). The functionally predicted impact of the missense variant was evaluated by MutationTaster (https://www.mutationtaster.org/), SIFT (Sortin Intolerant from Tolerant, https://sift.bii.a-star.edu.sg/), Polyphen2 (Polymorphism Phenotyping v2, http://genetics.bwh.harvard.edu/pph2/), REVEL (Rare Exome Variant Ensemble Learner, https://sites.google.com/site/revelgenomics/) and ClinPred (https://sites.google.com/site/clinpred/). Furthermore, the variants identified from proband were confirmed by Sanger sequencing on herself and her parents using Applied Biosystems ABI 3730xl sequencer, the primer sequences were as follows: *SETX* forward (5′-GGCCAAGATTGCACCAAGAT-3′) and reverse (5′-AACTGAGATCGCGCCACT-3′); *MME* forward (5′-GGTTGTGACTGAGACCTGTCAA-3′) and reverse (5′-AGTAAGCGTGAGCGCACTA-3′). The pathogenicity of new variants was classified according to the American College of Medical Genetics and Genomics/Association for Molecular Pathology (ACMG/AMP) guidelines (Richards et al., 2015).

### In silico analysis

To analyze the evolutionary conservation of *SETX* candidate variant, the gene and protein sequences of nine different species were derived from HomoloGene database (https://www.ncbi.nlm.nih.gov/homologene/?term=) and the alignment was performed by DNAMAN software (Version 9), including *Homo sapiens* (NM_015046.7, NP_055861.3), *Pan troglodytes* (XM_520331.4, XP_520331.2), *Macaca mulatta* (XM_002799947.1, XP_002799993.1), *Canis lupus familiaris* (XM_005625198.1, XP_005625255.1), *Bos taurus* (XM_002691649.3, XP_002691695.3), *Mus musculus* (NM_198033.2, NP_932150.2), *Rattus norvegicus* (XM_006233886.1, XP_006233948.1), *Gallus gallus* (XM_004945911.1, XP_004945968.1) and *Danio rerio* (XM_685853.6, XP_690945.5). The potential influence of the detected variant on the secondary structure of senataxin was evaluated by SOPMA (Self-Optimized Prediction method with Alignment, https://npsa-prabi.ibcp.fr/cgi-bin/npsa_automat.pl?page=npsa_sopma.html), and the three-dimensional (3D) structure was determined by SWISS-Model (https://swissmodel.expasy.org/) and visualized by PyMOL (Python-enhanced molecular graphics system) software (Version 2.6.0).

## Results

### Clinical manifestation of the proband

The proband (II-1) was a 40-year-old Chinese woman born to a non-consanguineous couple. She had normal developmental milestones, and her family history was unremarkable. Her lower limbs started to numb and weaken at the age of 16, and then she dropped out of school at the senior high school stage. The patient subsequently obtained employment in an optical factory, and her hips became numb and immobile after long periods of sitting. Later, she developed progressive gait disturbance and muscle atrophy in both lower extremities.

Physical and neurological examinations were performed at our hospital when the patient was 40 years old and revealed gait disturbances, cerebellar ataxia, peripheral neuropathy, hand dystonia, severe pes cavus and early-onset menopause but no oculomotor apraxia, head tremor or dysarthria. Sensory and motor examinations revealed absent tendon reflexes, deep sensory loss, muscular atrophy of both lower limbs and significant muscle weakness in the distal upper and lower limbs. The patient demonstrated no obvious cognitive impairment, with MMSE and MoCA scores of 27 (normal > 26 points) and 23 points (normal ≥ 25 points), respectively. Moreover, the patient required assistance with walking.

**Figure 1:**
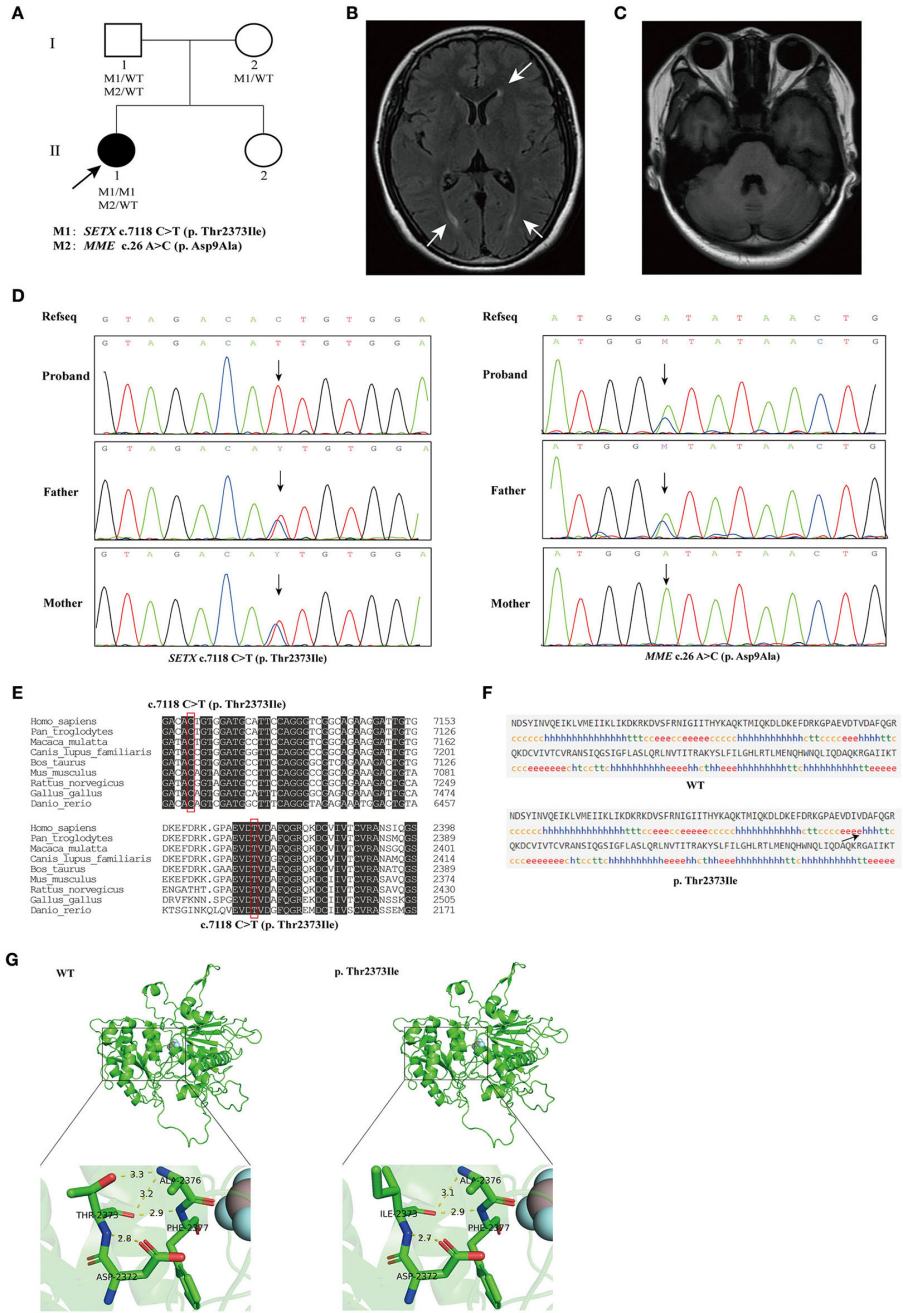
**(A)** The pedigree of the family. The black arrow indicates the proband that subjected for Whole-exome sequencing (WES). **(B)** Brain magnetic resonance imaging (MRI) of the proband, showing mild degeneration of white matter (white arrows). **(C)** Brain MRI of the proband, showing normal cerebellum. **(D)** Sequencing results showed that the homozygous variant c.7118C>T (p. Thr2373Ile) of *SETX* was inherited from both of the proband's parents (left), and the c.26A>C (p. Asp9Ala) variant in *MME* was inherited from the proband's father (right). **(E)** Conservation analysis of *SETX* c.7118C>T (p. Thr2373Ile) variant. **(F)** Secondary structures of wild-type and *SETX* p. Thr2373Ile variant were analyzed by SOPMA (Self-Optimized Prediction method with Alignment) online. h, α-helix; e, extended strand, arrow indicates change in secondary structure. **(G)** Prediction of the change in the three-dimensional (3D) structure of senataxin by *SETX* c.7118C>T (p. Thr2373Ile) variant.

Brain magnetic resonance imaging (MRI) showed a few lacunar foci in the subcortex of bilateral frontal lobe and mild degeneration of white matter, while without cerebellar and brainstem atrophy (Figures 1B,C). Nerve conduction studies (NCSs) showed that the compound muscle action potential (CMAP) amplitudes of partial motor nerves were decreased and that sensory nerve action potentials (SNAPs) were absent. Electromyography (EMG) showed multiple peripheral nerve damages involving some nerves of the upper and lower limbs and motor sensory axonal changes with myelin sheath damage. The laboratory assays indicated elevated serum AFP at 9.15 ng/mL (normal range <7.0 ng/ml), creatine kinase (CK) at 188 U/L (normal range 26–140 U/L), lipoprotein (a) at 696 mg/L (normal range <300 mg/L) and high-density lipoprotein cholesterol (HDL-C) at 1.68 mmol/L (normal range 1.03–1.55 mmol/L), while the levels of albumin, total cholesterol and immunoglobulins were normal. In addition, the serum sex hormone test showed that serum follicle-stimulating hormone (FSH) and luteinizing hormone (LH) were 39.41 and 15.7, respectively (interpreted as postmenopausal) and a decreased level of anti-Müllerian hormone (AMH) at 0.030 ng/ml (normal range 0.147–7.490 ng/ml). The clinical characteristics, laboratory indicators and molecular findings of the proband are shown in Table 1.

**Table 1:** Clinical and molecular characteristics of patients with SETX p. Thr2373 mutation.

**Variables**	**Current study**	**Bernard et al. (2008)**
**Sex/Age/Age at onset (year)**	F/40/16	F/21/17
**Initial symptom**	Lower limb weakness	Ataxia
**Clinical symptoms**		
Cerebellar ataxia	Yes	Yes
Oculomotor apraxia	No	No
Deep Tendon reflexes	Absent	No
Distal weakness	Yes	N/A
Deep sensory loss	Yes	N/A
Muscle atrophy	Lower limbs	No
Dystonia	Yes	Yes
Head tremor	No	N/A
Pes cavus	Yes	N/A
Cognitive impairment	No	No
Others	Menopause	Mild spastic paraparesis
**Neurophysiology**		
Peripheral neuropathy	Yes	Yes
Nerve conduction study	Sensorimotor neuropathy	N/A
**Brain MRI**		
Cerebellar atrophy	No	Yes
Leukoaraiosis	Mild	No
**Biochemistry**		
AFP (ng/ml)	9.15 (<7 ng/ml)	34 (<5 ng/ml)
CK (U/L)	188 (26–140 U/L)	263 (<171 U/L)
HDL-C (mmol/L)	1.68 (1.03–1.55 mmol/L)	N/A
Lp (a) (mg/L)	696 (<300 mg/L)	N/A
FSH (mIU/ml)	39.41 (Postmenopausal: 26.72–133.41 mIU/ml)	N/A
LH (mIU/ml)	15.7 (Postmenopausal: 5.61–61.99 mIU/ml)	N/A
AMH (ng/ml)	0.030 (0.147–7.490 ng/ml)	N/A
**Mutation**		
Nucleotide changes	c.7118C>T	c.6620A > T/c.7117A > C
Protein change	p. Thr2373Ile	p. Asp2207Val/p. Thr2373Pro
Mutation type	Homozygous	Compound heterozygous

AFP, α-fetoprotein; CK, creatine kinase; HDL-C, High-density lipoprotein cholesterol; Lp(a), Lipoprotein (a); FSH, Follicle-stimulating hormone; LH, Luteinizing hormone; AMH, anti-Müllerian hormone; N/A, not available.

### Genetic findings

The pedigree of the family is presented in Figure 1A. We performed next-generation sequencing (NGS) analysis for the proband. As a result, a novel homozygous missense variant in the *SETX* gene (NM_015046.7, exon 24, c.7118 C>T) and a heterozygous missense variant in the *MME* gene (NM_007289.3, exon 2, c.26 A>C) were identified. The c.7118 C>T variant in *SETX* caused a change of amino acid 2373 from threonine to isoleucine (p. Thr2373Ile), and the c.26 A>C variant in *MME* caused a change of amino acid 9 from aspartic acid to alanine (p. Asp9Ala). Sanger sequencing was performed to verify the variants on her parents (Figure 1D). The result indicated that both her parents were heterozygous carriers for the c.7118 C>T (p. Thr2373Ile) variant in the *SETX* gene, and her father was heterozygous for the c.26 A>C (p. Asp9Ala) variant in *MME*. However, the parents did not show any *SETX*- or *MME*-caused manifestations.

Heterozygous mutation in *MME* are associated with the Charcot–Marie–Tooth disease type 2T (CMT2T) (OMIM 617017) and spinocerebellar ataxia 43 (SCA43) (OMIM 617018), which both have a late-onset age and neither of them has symptoms of early menopause (Higuchi et al., 2016). The proband's father (I-1) was heterozygous for the *MME* c.26A > C variant and was not affected. The *MME* variant was of uncertain significance according to the ACMG/AMP guidelines as the criteria for pathogenic and benign are contradictory (BS4, PM2). Evaluating the clinical symptoms and family history, we considered that the c.26 A>C (p. Asp9Ala) variant in the *MME* gene may not be pathogenic in the proband in this study.

The homozygous or compound heterozygous mutations in *SETX* are associated with AOA2. The *SETX* p. Thr2373Ile variant has not been reported in the gnomAD, ClinVar and HGMD databases. Furthermore, no patient with the variant has been reported to date. The variant had a REVEL score of 0.948 and a ClinPred score of 0.9992; MutationTaster, SIFT and Polyphen2 predicted that this novel variant in the *SETX* gene was pathogenic. Conservative analysis showed that a threonine at position 2373 of *SETX* was evolutionarily highly conserved among various species (Figure 1E). The secondary structure of *SETX* was changed by p. Thr2373Ile variant (Figure 1F). Moreover, the three-dimensional (3D) protein structure models showed that one of the hydrogen bonds formed between Thr2373 and Ala2376 disappeared after Thr2373Ile variant, which may affect the protein spatial structure (Figure 1G). Additionally, an AOA2 patient with compound heterozygous mutations of c.6620 A > T (p. Asp2207Val) and c.7117 A>C (p. Thr2373Pro) in *SETX* gene was reported previously, one variant site was adjacent to c.7118 C>T and encoded the same amino acid 2373 (Table 1) (Bernard et al., 2008). According to the ACMG/AMP guidelines, the *SETX* c.7118 C>T variant was classified as likely pathogenic (PP3+PP4+PM2+PM5). Combining the clinical manifestations and genetic findings of the proband, we considered the proband as an AOA2 caused by a novel homozygous *SETX* missense mutation.

## Discussion

AOA2 is characterized by progressive cerebellar ataxia associated with peripheral neuropathy, cerebellar atrophy and elevated AFP concentrations (Anheim et al., 2009). In the present study, we report an AOA2 patient with a novel homozygous *SETX* mutation c.7118 C>T (p. Thr2373Ile). The patient's age of onset was 16 years, when she presented with numbness and weakness of the lower limbs. Then, she developed progressive gait disturbance accompanied by muscle atrophy of the lower limb extremities. She was characterized by cerebellar ataxia, hand dystonia, severe pes cavus, absent tendon reflexes, deep sensory loss and early-onset menopause. The pathogenicity of the *SETX* variant was confirmed by clinical symptoms, human genome variation database analysis and functional prediction. According to the ACMG guidelines, the *SETX* variant was categorized as a likely pathogenic variant.

Senataxin contains an N-terminal protein interaction domain and a DNA/RNA helicase in the C-terminal and is implicated in transcription, RNA processing, DNA damage responses and neurodevelopment (Yuce and West, 2013; Groh et al., 2017; Richard et al., 2021). Richard et al. (2021) found that *SETX* knockdown reduced transcripts encoding RNA-binding proteins and caused the accumulation of defective mitochondria and protein aggregates. Pathogenic variants of *SETX* cause two neurological diseases, AOA2, an autosomal recessive disorder, and ALS4, an autosomal dominant motor neuron disorder (Hadjinicolaou et al., 2021). *SETX* gene mutations were first reported as a cause of AOA2 by Moreira et al. (2004), they described 15 affected families with onset ages of 10–22 years who presented with gait ataxia, sensory motor neuropathy, cerebellar atrophy and high AFP levels; 15 variants in the *SETX* gene were identified, comprising five missense mutations, four nonsense mutations and six frameshift mutations (Moreira et al., 2004).

**Figure 2:**
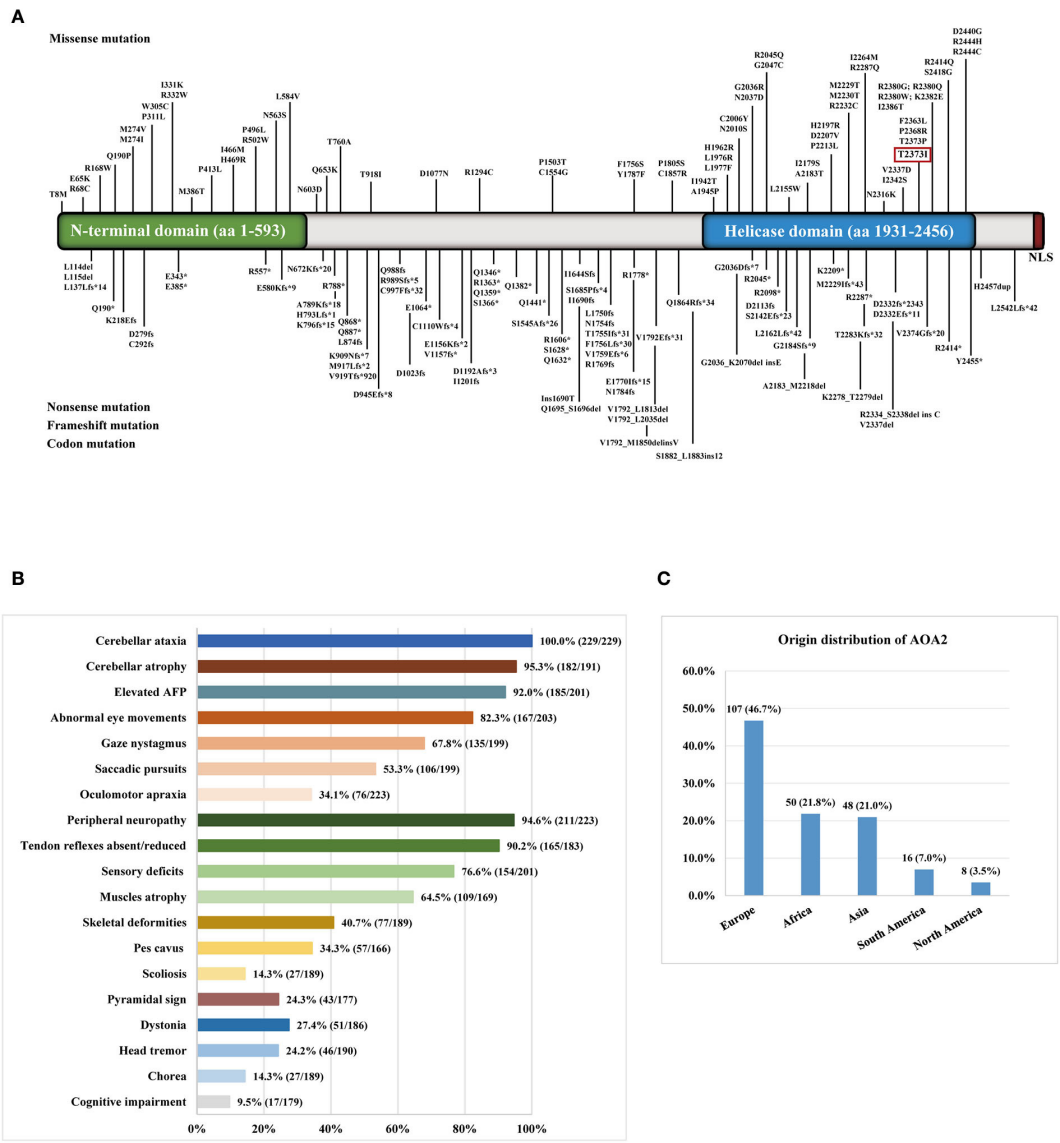
**(A)** Distribution of *SETX* gene variants in ataxia with oculomotor apraxia type 2 (AOA2) patients throughout exons. The variant in the red box was detected in the present study. **(B)** The clinical symptoms of AOA2 patients with *SETX* variants. **(C)** The origin distribution of the AOA2 patients.

We reviewed 57 studies on AOA2 patients with *SETX* variants conducted from 2004 to 2022 (Supplementary material). Among the variants, 156 homozygous or compound heterozygous variants in *SETX* genes were exonic (Figure 2A), comprising 70 missense mutations, 24 nonsense mutations, 57 frameshift mutations and 5 codon mutations. Among these mutations, 59 were located in the DNA/RNA helicase domain. Four studies were excluded because the patients' clinical features were unclear. Finally, we summarized the main clinical manifestations of 229 affected patients with *SETX* variants. The origin distribution of the patients is shown in Figure 2C. Nearly half of the patients (107, 46.7%) were European, 50 (21.8%) were African, 48 (21.0%) were Asian and 24 were American. Among the Asian patients, most of them were Pakistani (13 cases) and Japanese (11 cases), while 5 cases from two families were Mainland Chinese. The main clinical symptoms included cerebellar ataxia (100%), peripheral neuropathy (94.6%), cerebellar atrophy (95.3%), elevated AFP concentration (92.0%), oculomotor apraxia (OMA) (34.1%) and pes cavus (34.3%), while dystonia (27.4%), head tremor (24.2%), chorea (14.3%) and cognitive impairment (9.5%) were less frequent (Figure 2B). Furthermore, eight female patients had early menopause, and one had polycystic ovarian syndrome, two male patients had azoospermia and were infertile.

Our patient showed several clinical symptoms, including cerebellar ataxia, peripheral neuropathy, hand dystonia, pes cavus and elevated AFP, without oculomotor apraxia (OMA) and cerebellar atrophy. As reported, oculomotor apraxia (OMA) is not a basic feature in AOA2 patients. For example, Tazir et al. (2009) reported 19 patients with *SEXT* variants, all assessed patients had ataxia and elevated AFP concentrations, 80% of the affected patients presented with cerebellar dysarthria, 90% had sensory motor neuropathy, including muscular weakness and amyotrophy of distal lower limbs, and only 32% of the patients showed OMA. Nanetti et al. (2013) described 13 Italian patients who carried *SETX* variants. All these patients presented with gait unsteadiness, dysarthria, nystagmus, slow saccades, sensorimotor neuropathy and cerebellar atrophy of the vermis. Twelve patients had pes cavus, while none had symptoms of OMA and cognitive impairment (Nanetti et al., 2013). Duquette et al. (2005) studied 23 French-Canadian-affected AOA2 patients, who shared symptoms of gait ataxia, dysarthria, distal amyotrophy and saccadic pursuit, while none had OMA.

To the best of our knowledge, there were few reports of AOA2 patients in Mainland China. In 2016, two affected cases in one family from China with compound heterozygous variants, c.3190 G>T (p. E1064^*^) and c.4883 C>G (p. S1628^*^) of the *SETX* gene were identified, and both patients had early onset of unsteady gait, dysarthria, sensorimotor neuropathy, elevated serum AFP level and cerebellar atrophy (Lu et al., 2016). We observed a novel missense variant c.7118 C>T (p. Thr2373Ile) in exon 24 of the *SETX* gene on the proband, the variant identified in the present study affects the DNA/RNA helicase domain and may cause damage to helicase function, and the affected amino acid was fully conserved among several species. The C-terminal DNA/RNA helicase domain of the protein ranges from 1931 to 2456 amino acids. Studies have shown that variants located in the domain may cause AOA2 through the loss of function of senataxin (Ghrooda et al., 2012; Davis et al., 2013). Bernard et al. reported an AOA2 patient with compound heterozygosity of c.6620 A>T (p. Asp2207Val) and c.7117 A>C (p. Thr2373Pro) in *SETX* gene, the mutation site (c.7117 A>C) was adjacent to c.7118 C>T and encoded the same amino acid at position 2373, enhancing the possibility that the variant in this study may pathogenic. She developed ataxia at 17 years of age and clinical symptoms including cerebellar ataxia, mild spastic paraparesis, neuropathy, cerebellum atrophy and elevated serum AFP, without oculomotor apraxia, dystonia and cognitive impairment (Bernard et al., 2008).

Moreover, the present patient also experienced early menopause at 39 years old due to hypogonadism with FSH and LH levels in the postmenopausal range and low anti-Müllerian hormone (AMH) levels. Several previous studies have revealed that *SETX* may be related to hypogonadism, spermatogenesis and premature ovarian failure. The major clinical symptoms of nine previously reported AOA2 patients, who were affected by premature ovarian failure or azoospermia, are summarized in Supplementary Table S1. Subramanian et al. showed that *SETX* plays an important role in preventing premature ovarian failure in mice by maintaining the integrity of oocyte DNA. They found that *SETX* deficiency leads to an early-onset decline in female fertility by affecting DNA damage, a premature decline in ovarian follicle numbers and impaired meiotic maturation (Subramanian et al., 2021). Lynch et al. (2007) reported the case of a 21-year-old woman with primary ovarian failure, and her levels of serum FSH and LH indicated that she was postmenopausal; the homozygous mutation of *SETX* c.6292 C>T (p. Arg2098^*^) was then confirmed in the patient (Lynch et al., 2007). Mancini et al. (2015) reported the cases of three sisters from an Italian family with the *SETX* variant p. Arg2098^*^ who had all experienced early menopause in their thirties. These patients also demonstrated severe ataxia, saccadic pursuit, sensory motor neuropathy, kyphoscoliosis, head/hand tremor, chorea/myoclonia and vermian atrophy (Mancini et al., 2015). Gazulla et al. (2009) reported the case of a 37-year-old woman with AOA2 with a Val919Thrfs^*^920 homozygous variant in *SETX*. She had been experiencing recurrent amenorrhea for several years, with decreased 17-estradiol and elevated FSH, which was consistent with postmenopausal levels (Gazulla et al., 2009). Furthermore, two AOA2 cases with compound heterozygous variants of p. Met917Leufs^*^2/p. Met2230Thr and homozygous variant of p. Ser2142Glufs^*^23 in *SETX* genes were reported, and both had azoospermia and primary infertility. The role of *SETX* in azoospermia and infertility was subsequently determined by histopathological examinations of testes from AOA2 patients and *SETX* knockdown mice (Becherel et al., 2019; Catford et al., 2019).

However, there were several limitations of the present study. As the patient had not sought medical treatment at our hospital before, her past medical history and examination results were unclear, and the progression of her clinical symptoms was different to determine. Although we considered the *MME* p. Asp9Ala variant was not the cause of the patient's disease based on her onset age, clinical symptoms and family history, more family verification and functional experiments are needed to confirm this finding. Although the pathogenicity of the novel *SETX* variant identified is explained by the clinical phenotype of the proband, her onset age, the pedigree analysis and bioinformatic predictions, further functional experiments are needed to determine the impact of the variant on the expression and function of senataxin.

In conclusion, we identified a novel homozygous mutation of the *SETX* gene in a Chinese woman. Our report may expand the clinical and genetic spectrum of AOA2. Moreover, the clinical features of AOA2 and genetic findings in *SETX* were assessed from reported cohorts and are summarized in the present study.

## Data availability statement

The original contributions presented in the study are included in the article/Supplementary material and the sequencing data presented in the study are deposited in the Sequence Read Archive repository, accession number SRR21116234.

## Ethics statement

The studies involving human participants were reviewed and approved by the Ethics Committee of Taizhou Hospital of Zhejiang Province Affiliated to Wenzhou Medical University. The patients/participants provided their written informed consent to participate in this study. Written informed consent was obtained from the individual(s) for the publication of any potentially identifiable images or data included in this article.

## Author contributions

ShuC, JD, and YZ conceived the study. ShuC analyzed the data and wrote the manuscript. YZ and HJ provided the clinical samples. AY and WZ participated in the clinical data collection and clinical evaluations. ShuC, JD, NW, and BS performed the genetic analysis and bioinformatics analysis. ShiC and JL conducted the biochemical analysis. BS and YZ critically commented and revised the manuscript. All authors have read and approved the final version of the manuscript.

## Conflict of interest

The authors declare that the research was conducted in the absence of any commercial or financial relationships that could be construed as a potential conflict of interest.

## Publisher's note

All claims expressed in this article are solely those of the authors and do not necessarily represent those of their affiliated organizations, or those of the publisher, the editors and the reviewers. Any product that may be evaluated in this article, or claim that may be made by its manufacturer, is not guaranteed or endorsed by the publisher.
