# Supplementary material for: Ataxia with oculomotor apraxia type 2 caused by a novel homozygous mutation in SETX gene, and literature review
Source: Front Mol Neurosci. 2022 Nov 10;15:1019974. doi: 10.3389/fnmol.2022.1019974 (PMC9684320; doi:10.3389/fnmol.2022.1019974)
Supplement: Supplementary file 1 [file Data_Sheet_1.docx]

**Supplementary material: 57 literatures about AOA2 patients with SETX mutations from 2004-2022**

[1] Moreira M.C., Klur S., Watanabe M., Nemeth A.H., Le Ber I., Moniz J.C., et al. Senataxin, the ortholog of a yeast RNA helicase, is mutant in ataxia-ocular apraxia 2. Nature genetics 36 (2004) 225-7. doi. 10.1038/ng1303

[2] Asaka T., Yokoji H., Ito J., Yamaguchi K., and Matsushima A. Autosomal recessive ataxia with peripheral neuropathy and elevated AFP: novel mutations in SETX. Neurology 66 (2006) 1580-1. doi. 10.1212/01.wnl.0000216135.59699.9b

[3] Duquette A., Roddier K., McNabb-Baltar J., Gosselin I., St-Denis A., Dicaire M.J., et al. Mutations in senataxin responsible for Quebec cluster of ataxia with neuropathy. Ann Neurol 57 (2005) 408-14. doi. 10.1002/ana.20408

[4] Criscuolo C., Chessa L., Di Giandomenico S., Mancini P., Sacca F., Grieco G.S., et al. Ataxia with oculomotor apraxia type 2: a clinical, pathologic, and genetic study. Neurology 66 (2006) 1207-10. doi. 10.1212/01.wnl.0000208402.10512.4a

[5] Chen Y.Z., Hashemi S.H., Anderson S.K., Huang Y., Moreira M.C., Lynch D.R., et al. Senataxin, the yeast Sen1p orthologue: characterization of a unique protein in which recessive mutations cause ataxia and dominant mutations cause motor neuron disease. Neurobiol Dis 23 (2006) 97-108. doi. 10.1016/j.nbd.2006.02.007

[6] Bassuk A.G., Chen Y.Z., Batish S.D., Nagan N., Opal P., Chance P.F., et al. In cis autosomal dominant mutation of Senataxin associated with tremor/ataxia syndrome. Neurogenetics 8 (2007) 45-9. doi. 10.1007/s10048-006-0067-8

[7] Lynch D.R., Braastad C.D., and Nagan N. Ovarian failure in ataxia with oculomotor apraxia type 2. American journal of medical genetics. Part A 143A (2007) 1775-7. doi. 10.1002/ajmg.a.31816

[8] Schols L., Arning L., Schule R., Epplen J.T., and Timmann D. "Pseudodominant inheritance" of ataxia with ocular apraxia type 2 (AOA2). J Neurol 255 (2008) 495-501. doi. 10.1007/s00415-008-0707-z

[9] Arning L., Schols L., Cin H., Souquet M., Epplen J.T., and Timmann D. Identification and characterisation of a large senataxin (SETX) gene duplication in ataxia with ocular apraxia type 2 (AOA2). Neurogenetics 9 (2008) 295-9. doi. 10.1007/s10048-008-0139-z

[10] Nicolaou P., Georghiou A., Votsi C., Middleton L.T., Zamba-Papanicolaou E., and Christodoulou K. A novel c.5308_5311delGAGA mutation in Senataxin in a Cypriot family with an autosomal recessive cerebellar ataxia. BMC Med Genet 9 (2008) 28. doi. 10.1186/1471-2350-9-28

[11] Bernard V., Stricker S., Kreuz F., Minnerop M., Gillessen-Kaesbach G., and Zuhlke C. Ataxia with oculomotor apraxia type 2: novel mutations in six patients with juvenile age of onset and elevated serum alpha-fetoprotein. Neuropediatrics 39 (2008) 347-50. doi. 10.1055/s-0029-1214424

[12] Anheim M., Fleury M.C., Franques J., Moreira M.C., Delaunoy J.P., Stoppa-Lyonnet D., et al. Clinical and molecular findings of ataxia with oculomotor apraxia type 2 in 4 families. Arch Neurol 65 (2008) 958-62. doi. 10.1001/archneur.65.7.958

[13] Tazir M., Ali-Pacha L., M'Zahem A., Delaunoy J.P., Fritsch M., Nouioua S., et al. Ataxia with oculomotor apraxia type 2: a clinical and genetic study of 19 patients. J Neurol Sci 278 (2009) 77-81. doi. 10.1016/j.jns.2008.12.004

[14] Gazulla J., Benavente I., Lopez-Fraile I.P., Modrego P., and Koenig M. Sensorimotor neuronopathy in ataxia with oculomotor apraxia type 2. Muscle Nerve 40 (2009) 481-5. doi. 10.1002/mus.21328

[15] Nakamura K., Yoshida K., Makishita H., Kitamura E., Hashimoto S., and Ikeda S. A novel nonsense mutation in a Japanese family with ataxia with oculomotor apraxia type 2 (AOA2). J Hum Genet 54 (2009) 746-8. doi. 10.1038/jhg.2009.104

[16] Fogel B.L., Lee J.Y., and Perlman S. Aberrant splicing of the senataxin gene in a patient with ataxia with oculomotor apraxia type 2. Cerebellum 8 (2009) 448-53. doi. 10.1007/s12311-009-0130-8

[17] Airoldi G., Guidarelli A., Cantoni O., Panzeri C., Vantaggiato C., Bonato S., et al. Characterization of two novel SETX mutations in AOA2 patients reveals aspects of the pathophysiological role of senataxin. Neurogenetics 11 (2010) 91-100. doi. 10.1007/s10048-009-0206-0

[18] Bernard V., Minnerop M., Burk K., Kreuz F., Gillessen-Kaesbach G., and Zuhlke C. Exon deletions and intragenic insertions are not rare in ataxia with oculomotor apraxia 2. BMC Med Genet 10 (2009) 87. doi. 10.1186/1471-2350-10-87

[19] H'Mida-Ben Brahim D., M'Zahem A., Assoum M., Bouhlal Y., Fattori F., Anheim M., et al. Molecular diagnosis of known recessive ataxias by homozygosity mapping with SNP arrays. J Neurol 258 (2011) 56-67. doi. 10.1007/s00415-010-5682-5

[20] Gazulla J., Benavente I., Lopez-Fraile I.P., Tordesillas C., Modrego P., Alonso I., et al. Sensory neuronopathy in ataxia with oculomotor apraxia type 2. J Neurol Sci 298 (2010) 118-20. doi. 10.1016/j.jns.2010.09.004

[21] Bohlega S.A., Shinwari J.M., Al Sharif L.J., Khalil D.S., Alkhairallah T.S., and Al Tassan N.A. Clinical and molecular characterization of ataxia with oculomotor apraxia patients in Saudi Arabia. BMC Med Genet 12 (2011) 27. doi. 10.1186/1471-2350-12-27

[22] Hammer M.B., El Euch-Fayache G., Nehdi H., Saidi D., Nasri A., Nabli F., et al. Clinical and molecular findings of ataxia with oculomotor apraxia type 2 (AOA2) in 5 Tunisian families. Diagn Mol Pathol 21 (2012) 241-5. doi. 10.1097/PDM.0b013e318257ad9a

[23] Davis M.Y., Keene C.D., Swanson P.D., Sheehy C., and Bird T.D. Novel mutations in ataxia telangiectasia and AOA2 associated with prolonged survival. J Neurol Sci 335 (2013) 134-8. doi. 10.1016/j.jns.2013.09.014

[24] Ichikawa Y., Ishiura H., Mitsui J., Takahashi Y., Kobayashi S., Takuma H., et al. Exome analysis reveals a Japanese family with spinocerebellar ataxia, autosomal recessive 1. J Neurol Sci 331 (2013) 158-60. doi. 10.1016/j.jns.2013.05.018

[25] Nanetti L., Cavalieri S., Pensato V., Erbetta A., Pareyson D., Panzeri M., et al. SETX mutations are a frequent genetic cause of juvenile and adult onset cerebellar ataxia with neuropathy and elevated serum alpha-fetoprotein. Orphanet J Rare Dis 8 (2013) 123. doi. 10.1186/1750-1172-8-123

[26] Datta N., and Hohler A. A new SETX mutation producing AOA2 in two siblings. The International journal of neuroscience 123 (2013) 670-3. doi. 10.3109/00207454.2013.787616

[27] Vantaggiato C., Cantoni O., Guidarelli A., Romaniello R., Citterio A., Arrigoni F., et al. Novel SETX variants in a patient with ataxia, neuropathy, and oculomotor apraxia are associated with normal sensitivity to oxidative DNA damaging agents. Brain Dev 36 (2014) 682-9. doi. 10.1016/j.braindev.2013.10.003

[28] Brugger F., Schupbach M., Koenig M., Muri R., Bohlhalter S., Kaelin-Lang A., et al. The Clinical Spectrum of Ataxia with Oculomotor Apraxia Type 2. Mov Disord Clin Pract 1 (2014) 106-109. doi. 10.1002/mdc3.12021

[29] Roda R.H., Rinaldi C., Singh R., Schindler A.B., and Blackstone C. Ataxia with oculomotor apraxia type 2 fibroblasts exhibit increased susceptibility to oxidative DNA damage. J Clin Neurosci 21 (2014) 1627-31. doi. 10.1016/j.jocn.2013.11.048

[30] Pera J., Lechner S., Biskup S., Strach M., Grodzicki T., and Slowik A. Two novel mutations of the SETX gene and ataxia with oculomotor apraxia type 2. Clin Neurol Neurosurg 128 (2015) 44-6. doi. 10.1016/j.clineuro.2014.10.024

[31] Mancini C., Orsi L., Guo Y., Li J., Chen Y., Wang F., et al. An atypical form of AOA2 with myoclonus associated with mutations in SETX and AFG3L2. BMC Med Genet 16 (2015) 16. doi. 10.1186/s12881-015-0159-0

[32] Newrick L., Taylor M., and Hadjivassiliou M. Pseudodominant AOA2. Cerebellum Ataxias 2 (2015) 5. doi. 10.1186/s40673-015-0024-0

[33] Lu C., Zheng Y.C., Dong Y., and Li H.F. Identification of novel senataxin mutations in Chinese patients with autosomal recessive cerebellar ataxias by targeted next-generation sequencing. BMC neurology 16 (2016) 179. doi. 10.1186/s12883-016-0696-y

[34] Motokura E., Yamashita T., Takahashi Y., Tsunoda K., Sato K., Takemoto M., et al. An AOA2 patient with a novel compound heterozygous SETX frame shift mutations. J Neurol Sci 372 (2017) 294-296. doi. 10.1016/j.jns.2016.11.074

[35] Mariani L.L., Rivaud-Pechoux S., Charles P., Ewenczyk C., Meneret A., Monga B.B., et al. Comparing ataxias with oculomotor apraxia: a multimodal study of AOA1, AOA2 and AT focusing on video-oculography and alpha-fetoprotein. Scientific reports 7 (2017) 15284. doi. 10.1038/s41598-017-15127-9

[36] Tariq H., Imran R., and Naz S. A Novel Homozygous Variant of SETX Causes Ataxia with Oculomotor Apraxia Type 2. J Clin Neurol 14 (2018) 498-504. doi. 10.3988/jcn.2018.14.4.498

[37] Kashimada A., Hasegawa S., Nomura T., Shiraku H., Moriyama K., Suzuki T., et al. Genetic analysis of undiagnosed ataxia-telangiectasia-like disorders. Brain Dev 41 (2019) 150-157. doi. 10.1016/j.braindev.2018.09.007

[38] Paucar M., Taylor A.M.R., Hadjivassiliou M., Fogel B.L., and Svenningsson P. Progressive Ataxia with Elevated Alpha-Fetoprotein: Diagnostic Issues and Review of the Literature. Tremor Other Hyperkinet Mov (N Y) 9 (2019). doi. 10.7916/tohm.v0.708

[39] Becherel O.J., Fogel B.L., Zeitlin S.I., Samaratunga H., Greaney J., Homer H., et al. Disruption of Spermatogenesis and Infertility in Ataxia with Oculomotor Apraxia Type 2 (AOA2). Cerebellum 18 (2019) 448-456. doi. 10.1007/s12311-019-01012-w

[40] Catford S.R., O'Bryan M.K., McLachlan R.I., Delatycki M.B., and Rombauts L. Germ cell arrest associated with aSETX mutation in ataxia oculomotor apraxia type 2. Reprod Biomed Online 38 (2019) 961-965. doi. 10.1016/j.rbmo.2018.12.042

[41] Perry M.D., Evans M.J., Byrd P.J., and Taylor M.R. Biallelic Mutation of SETX and Additional Likely "In Cis" SETX Sequence Change in Ataxia with Oculomotor Apraxia Type 2. J Pediatr Genet 10 (2021) 311-314. doi. 10.1055/s-0040-1713909

[42] Tariq H., Tariq I., Bourinaris T., Houlden H., and Naz S. Some pathogenic SETX variants are partially conserved during evolution. Gene 771 (2021) 145360. doi. 10.1016/j.gene.2020.145360

[43] Hadjinicolaou A., Ngo K.J., Conway D.Y., Provias J.P., Baker S.K., Brady L.I., et al. De novo pathogenic variant in SETX causes a rapidly progressive neurodegenerative disorder of early childhood-onset with severe axonal polyneuropathy. Acta Neuropathol Commun 9 (2021) 194. doi. 10.1186/s40478-021-01277-5

[44] Chang H.J., Kim R., Kim M., Moon J., Kim M.J., and Kim H.J. A Case of AOA2 With Compound Heterozygous SETX Mutations. J Mov Disord 15 (2022) 178-180. doi. 10.14802/jmd.21139

[45] Kinkar J.S., Jameel P.Z., Kumawat B.L., and Kalbhor P. Heterozygous deletion in exon 6 of STEX gene causing ataxia with oculomotor apraxia type 2 (AOA-2) with ovarian failure. BMJ Case Rep 14 (2021). doi. 10.1136/bcr-2021-241767

[46] da Costa S.C.G., de Rezende-Filho F.C., de Freitas J.L., de Assis Pereira Matos P.C.A., Della-Ripa B., Franca M.C., Jr., et al. Clinical and Genetic Characterization of Brazilian Patients with Ataxia and Oculomotor Apraxia. Mov Disord 37 (2022) 1309-1316. doi. 10.1002/mds.29015

[47] Chiang P.I., Liao T.W., and Chen C.M. A Novel SETX Mutation in a Taiwanese Patient with Autosomal Recessive Cerebellar Ataxia Detected by Targeted Next-Generation Sequencing, and a Literature Review. Brain Sci 12 (2022). doi. 10.3390/brainsci12020173

[48] Ponger P., Kurolap A., Lerer I., Dagan J., Chai Gadot C., Mory A., et al. Unique Ataxia-Oculomotor Apraxia 2 (AOA2) in Israel with Novel Variants, Atypical Late Presentation, and Possible Identification of a Poison Exon. J Mol Neurosci (2022). doi. 10.1007/s12031-022-02035-5

[49] Algahtani H., Shirah B., Algahtani R., Naseer M.I., Al-Qahtani M.H., and Abdulkareem A.A. Ataxia with ocular apraxia type 2 not responding to 4-aminopyridine: A rare mutation in the SETX gene in a Saudi patient. Intractable Rare Dis Res 7 (2018) 275-279. doi. 10.5582/irdr.2018.01107

[50] Szpisjak L., Obal I., Engelhardt J.I., Vecsei L., and Klivenyi P. A novel SETX gene mutation producing ataxia with oculomotor apraxia type 2. Acta Neurol Belg 116 (2016) 405-7. doi. 10.1007/s13760-015-0569-y

[51] Ghrooda S., Borys A., Spriggs E., Hegde M., and Mhanni A. SETX gene novel mutations in a non-French Canadian with ataxia-oculomotor apraxia type 2. Parkinsonism Relat Disord 18 (2012) 700-1. doi. 10.1016/j.parkreldis.2012.01.022

[52] Marelli C., Guissart C., Hubsch C., Renaud M., Villemin J.P., Larrieu L., et al. Mini-Exome Coupled to Read-Depth Based Copy Number Variation Analysis in Patients with Inherited Ataxias. Human mutation 37 (2016) 1340-1353. doi. 10.1002/humu.23063

[53] Anheim M., Monga B., Fleury M., Charles P., Barbot C., Salih M., et al. Ataxia with oculomotor apraxia type 2: clinical, biological and genotype/phenotype correlation study of a cohort of 90 patients. Brain 132 (2009) 2688-98. doi. 10.1093/brain/awp211

[54] Fogel B.L., Cho E., Wahnich A., Gao F., Becherel O.J., Wang X., et al. Mutation of senataxin alters disease-specific transcriptional networks in patients with ataxia with oculomotor apraxia type 2. Hum Mol Genet 23 (2014) 4758-69. doi. 10.1093/hmg/ddu190

[55] Radziwonik W., Elert-Dobkowska E., Klimkowicz-Mrowiec A., Ziora-Jakutowicz K., Stepniak I., Zaremba J., et al. Application of a custom NGS gene panel revealed a high diagnostic utility for molecular testing of hereditary ataxias. J Appl Genet (2022). doi. 10.1007/s13353-022-00701-3

[56] Zuhlke C., Bernard V., and Gillessen-Kaesbach G. Investigation of recessive ataxia loci in patients with young age of onset. Neuropediatrics 38 (2007) 207-9. doi. 10.1055/s-2007-990268

[57] Hao Y., Gu W.H., Chen Y.Y., and Zhang J. Homozygosity mapping and mutation analysis of a consanguineous marriage family with autosomal recessive cerebellar ataxia. Chinese Journal of Neurology (2015) 400-405. doi. 10.3760/cma.j.issn.1006-7876.2015.05.011
