# Supplementary material for: Ataxia with oculomotor apraxia type 2 caused by a novel homozygous mutation in SETX gene, and literature review
Source: Front Mol Neurosci. 2022 Nov 10;15:1019974. doi: 10.3389/fnmol.2022.1019974 (PMC9684320; doi:10.3389/fnmol.2022.1019974)
Supplement: Supplementary file 2 [file Table_1.DOCX]

**Table S1. Genotypic and phenotypic characteristics of the AOA2 patients with ovarian failure or azoospermia.**

|  | Lynch DR et al. (2007)[1] | Gazulla J et al. (2009)[2] | Fogel BL et al. (2009)[3] | Mancini C et al. (2015)[4] | Mancini C et al. (2015)[4] | Mancini C et al. (2015)[4] | Becherel OJ et al. (2019)[5] | Catford SR et al. (2019)[6] | Kinkar JS et al. (2021)[7] |
| --- | --- | --- | --- | --- | --- | --- | --- | --- | --- |
| Origin | Syria | Spain | Pakistan | Italian | Italian | Italian | Eastern European | Afghanistan | Indian |
| Sex/age/age at onset (y) | F/21/14 | F/37/25 | F/18/14 | F/51/12 | F/49/9 | F/44/12 | M/24/15 | M/25/18 | F/21/18 |
| Initial symptom | Slowly progressive ataxia and dyscoordination | Dysarthria | Imbalance, falls, and difficulty walking | Chorea/myoclonia | Chorea/myoclonia | Chorea/myoclonia | Slowly progressive ataxia | Unsteady gait and upper limb incoordination | Progressive difficulty in walking |
| Cerebellar ataxia | Yes | Yes | Yes | Yes | Yes | Yes | Yes | Yes | Yes |
| Dysarthria | Yes | Yes | Yes | NO | NO | NO | NA | N/A | Yes |
| Oculomotor apraxia | NO | Yes | NO | NO | NO | NO | NO | N/A | Yes |
| Other abnormal eye movements | Nystagmus | Nystagmus  Abnormal ocular pursuit | Slow saccades | Nystagmus  Saccadic pursuit | Nystagmus Saccadic pursuit | Nystagmus Saccadic pursuit  Strabismus | Nystagmus  Saccadic smooth pursuit | N/A | Nystagmus  Slow saccades Diplopia |
| Amyotrophy | NO | Yes | NO | Yes | Yes | Yes | Yes | N/A | NO |
| Tendon reflexes | Absent | Absent | Absent | Absent | Absent | Absent | Yes | N/A | Absent |
| Sensory impaired | Yes | Yes | Yes | Yes | Yes | Yes | Yes | N/A | Yes |
| Skeletal deformities | NO | NO | N/A | Kyphoscoliosis | Kyphoscoliosis | Kyphoscoliosis | N/A | N/A | NO |
| Pes cavus | NO | NO | N/A | NO | No | NO | N/A | N/A | NO |
| Head/hand tremor | NO | NO | N/A | Yes | Yes | Yes | N/A | N/A | NO |
| Choreic movements | NO | NO | N/A | Yes | Yes | Yes | N/A | N/A | Yes |
| Dystonia | Yes | NO | N/A | Yes | Yes | NO | N/A | N/A | Yes |
| Cognitive impairment | NO | NO | NO | NO | NO | Yes | NO | N/A | NO |
| Nerve  conduction study | N/A | Sensory and motor neuronopathy | Sensorimotor axonal neuropathy. | NO | NO | NO | N/A | N/A | Axonal sensorimotor neuropathy |
| Brain MRI | Cerebellar atrophy | Cerebellar atrophy | Cerebellar atrophy | Cerebellar atrophy | Cerebellar atrophy | Cerebellar atrophy/  Brainstem atrophy | N/A | N/A | Cerebellar atrophy |
| Elevated AFP | Yes | Yes | Yes | Yes | Yes | Yes | N/A | N/A | Yes |
| Others | Postmenopausal  Primary ovarian failure | Postmenopausal Primary ovarian failure | Polycystic ovarian syndrome | Early menopause | Early menopause | Early menopause | Azoospermia | Primary infertility and azoospermia | Menopausal  Premature ovarian failure |
| SETX mutations | c.6292C>T (p.Arg2098*) | 2755_2756delGT(fsVal919Thr920*) | IVS16+2insT | c.6292C>T (p.Arg2098*) | c.6292C>T (p.Arg2098*) | c.6292C>T (p.Arg2098*) | c.2747_2748insAT(p.Met917Leufs*2)/  c.6689T>C(p.Met2230Thr) | c.6422dup (p.Ser2142Glufs*23) | Exon 6 deleted |
| Mutation status | homozygous | homozygous | homozygous | homozygous | homozygous | homozygous | compound heterozygous | homozygous | heterozygous |

Note: N/A: Not performed/not available; NO: Normal; AFP: Alpha-fetoprotein

**Reference:**

[1] Lynch D.R., Braastad C.D., and Nagan N. Ovarian failure in ataxia with oculomotor apraxia type 2. American journal of medical genetics. Part A 143A (2007) 1775-7. doi. 10.1002/ajmg.a.31816

[2] Gazulla J., Benavente I., Lopez-Fraile I.P., Modrego P., and Koenig M. Sensorimotor neuronopathy in ataxia with oculomotor apraxia type 2. Muscle Nerve 40 (2009) 481-5. doi. 10.1002/mus.21328

[3] Fogel B.L., Lee J.Y., and Perlman S. Aberrant splicing of the senataxin gene in a patient with ataxia with oculomotor apraxia type 2. Cerebellum 8 (2009) 448-53. doi. 10.1007/s12311-009-0130-8

[4] Mancini C., Orsi L., Guo Y., Li J., Chen Y., Wang F., et al. An atypical form of AOA2 with myoclonus associated with mutations in SETX and AFG3L2. BMC Med Genet 16 (2015) 16. doi. 10.1186/s12881-015-0159-0

[5] Becherel O.J., Fogel B.L., Zeitlin S.I., Samaratunga H., Greaney J., Homer H., et al. Disruption of Spermatogenesis and Infertility in Ataxia with Oculomotor Apraxia Type 2 (AOA2). Cerebellum 18 (2019) 448-456. doi. 10.1007/s12311-019-01012-w

[6] Catford S.R., O'Bryan M.K., McLachlan R.I., Delatycki M.B., and Rombauts L. Germ cell arrest associated with aSETX mutation in ataxia oculomotor apraxia type 2. Reprod Biomed Online 38 (2019) 961-965. doi. 10.1016/j.rbmo.2018.12.042

[7] Kinkar J.S., Jameel P.Z., Kumawat B.L., and Kalbhor P. Heterozygous deletion in exon 6 of STEX gene causing ataxia with oculomotor apraxia type 2 (AOA-2) with ovarian failure. BMJ Case Rep 14 (2021). doi. 10.1136/bcr-2021-241767
